# A specific structure and high richness characterize intestinal microbiota of HIV-exposed seronegative individuals

**DOI:** 10.1371/journal.pone.0260729

**Published:** 2021-12-02

**Authors:** Tulio J. Lopera, Jorge A. Lujan, Eduardo Zurek, Wildeman Zapata, Juan C. Hernandez, Miguel A. Toro, Juan F. Alzate, Natalia A. Taborda, Maria T. Rugeles, Wbeimar Aguilar-Jimenez

**Affiliations:** 1 Facultad de Medicina, Grupo Inmunovirología, Universidad de Antioquia UdeA, Medellín, Colombia; 2 Department of System Engineering, Universidad del Norte, Barranquilla, Colombia; 3 Facultad de Medicina, Grupo Infettare, Universidad Cooperativa de Colombia, Medellín, Colombia; 4 Facultad de Medicina, Centro Nacional de Secuenciación Genómica -CNSG, Sede de Investigación Universitaria -SIU, Universidad de Antioquia UdeA, Medellin, Colombia; 5 Facultad de Medicina, Grupo de Parasitología, Universidad de Antioquia, Medellín, Colombia; 6 Facultad de Ciencias de la Salud, Grupo de Investigaciones Biomédicas Uniremington, Programa de Medicina, Corporación Universitaria Remington, Medellín, Colombia; Emory University School of Medicine, UNITED STATES

## Abstract

Intestinal microbiota facilitates food breakdown for energy metabolism and influences the immune response, maintaining mucosal homeostasis. Overall, HIV infection is associated with intestinal dysbiosis and immune activation, which has been related to seroconversion in HIV-exposed individuals. However, it is unclear whether microbiota dysbiosis is the cause or the effect of immune alterations and disease progression or if it could modulate the risk of acquiring the HIV infection. We characterize the intestinal microbiota and determine its association with immune regulation in HIV-exposed seronegative individuals (HESN), HIV-infected progressors (HIV+), and healthy control (HC) subjects. For this, feces and blood were collected. The microbiota composition of HESN showed a significantly higher alpha (p = 0.040) and beta diversity (p = 0.006) compared to HC, but no differences were found compared to HIV+. A lower Treg percentage was observed in HESN (1.77%) than HC (2.98%) and HIV+ (4.02%), with enrichment of the genus *Butyrivibrio* (p = 0.029) being characteristic of this profile. Moreover, we found that *Megasphaera* (p = 0.017) and *Victivallis* (p = 0.0029) also are enriched in the microbiota composition in HESN compared to HC and HIV+ subjects. Interestingly, an increase in *Succinivibrio* and *Prevotella*, and a reduction in *Bacteroides* genus, which is typical of HIV-infected individuals, were observed in both HESN and HIV+, compared to HC. Thus, HESNs have a microbiota profile, similar to that observed in HIV+, most likely because HESN are cohabiting with their HIV+ partners.

## Introduction

Resistance to Human Immunodeficiency Virus 1 (HIV-1) in uninfected individuals who have repeated unprotected sexual exposure to the virus, known as HIV-1-exposed seronegative (HESN), provides a unique opportunity to elucidate mechanisms of natural protection to infection. Although the mechanisms of protection in HESNs have not been fully elucidated, a potent but focused and tightly regulated innate antiviral response at the entry site, clearing the virus while avoiding excessive immune activation, is commonly observed in HESNs [1–4]. Furthermore, the gastrointestinal tract (GIT), as the primary viral replication site and viral reservoir for HIV-1, plays an essential role in viral control and eradication [5].

Recent studies demonstrated that gut microbiota, composed of over 100 trillion bacteria residing at the GIT, facilitates food breakdown for energy metabolism and contributes to the immune system’s development and function, maintaining mucosal homeostasis [5,6]. Modification of the intestinal microbiota composition is related to alterations in metabolic processes and some metabolic disorders [7,8].

In the HIV context, most studies, including those performed in Latin America, have found that HIV infection is associated with intestinal dysbiosis and changes in diversity, with increased numbers of *Enterobacteriaceae* and *Enterococcaceae* pathobionts, as well as by an enrichment of the *Prevotella* genus [9–12]. This altered microbial profile in HIV+ patients is associated with *i)* a metabolic alteration of the microbiota and processes related to oxidative stress [13], and with *ii)* increased levels of activation markers, and reduced levels of IL-10 and IL-1R, which in turn correlate with an increase of activated CD8^+^ T cells [9,14,15], possibly facilitating infection in HIV-exposed individuals [16]. Therefore, the presence and abundance of beneficial bacteria within the intestinal microbiota could modulate the development and response of the immune system, decrease activation levels, and promote integrity and function of the intestinal mucosa, consequently protecting against infections such as those caused by enteric pathogens and sexually transmitted viruses as HIV-1 [17,18].

Nevertheless, to date, it is not clear if the microbiota dysbiosis in HIV-infected individuals is a product of the immunological alterations induced by the virus or whether it is one of the causes of immune alterations, promoting disease progression. Even so, few studies have proposed to evaluate the relationship between the intestinal microbiota and susceptibility to this infection in HIV-exposed subjects. Some studies have found that men who have sex with men (MSM) have a richer and more diverse fecal microbiota than non-MSM people. However, in the HESN context, most studies have focused on women [19], especially in the cervicovaginal microbiota in cohorts of sex workers in Africa. These studies have associated protective mucosal immune responses with a highly diverse vaginal microbiota, where, for example, the presence of *Lactobacillus* species is related to a lower risk of HIV-1 transmission [20–22].

Hence, we characterize the intestinal microbiota, determining its association with natural resistance to HIV, as well as with immune activation and regulation in HESN, HIV+, and non-exposed HC from a small cohort of Colombian individuals.

## Materials and methods

### Population

General inclusion criteria for the three groups of the study were: to be over 18 years old, have no history of recent antibiotic use, diarrhea, gastrointestinal disease or consumption of antiretroviral medication. Blood and stool samples were collected from 6 HESN from a serodiscordant cohort of heterosexual couples recruited at the HIV-1 care program HERES in Santa Marta, Colombia. Inclusion criteria for HESN were: seronegative at the time of inclusion, with a history of unprotected sexual intercourse with HIV positive partners with detectable viral loads, 12 or more unprotected sexual episodes in at least three consecutive months within one year of study entry. Also, a third-generation rapid test and the determination of proviral DNA were performed, obtaining negative results [23].

The HESN subjects were 33% males, mean age [range] = 37 [18–58] years old, and their HIV-1 seropositive partners had at sampling: i) viral load of 1,450 [50–180,790] copies/mL (2 cART naïve, one on suppressive cART, and three on cART with low adherence), ii) CD4^+^ T cells count of 344 [134–804] cells/uL and iii) the length of HIV infection since diagnosis was 9.5 [3.6–14.4] years. The couples had an average of 15 unprotected sexual intercourses per month during 5.5 [2.2–9.0] years, with the last unprotected intercourse taking place between two days and six months before sampling.

Blood and stool samples from 9 healthy controls (HC) with low risk for HIV infection were also included (45% males with mean age [range] = 25 [22–53] years). Additionally, blood and stool samples from 10 HIV-1 infected subjects were included (90% males with mean age [range] = 27 [18–43] years). They had evident disease progression based in next criteria: HIV-1 infection diagnostic (without antiretroviral treatment), CD4^+^ T-cells count >180 cells/μL and viral load between 7,000 and 100,000 copies/mL.

Most individuals in this study were heterosexual. Additionally, each subject was surveyed on diet components, the consumption of medications, and aspects related to lifestyle known modifiers of the intestinal microbiota [24,25]. Exclusion criteria were diarrhea or being on antibiotic treatment at the time of sampling. The study was designed and conducted following the Declaration of Helsinki and Colombia legislation (Ministry of health resolution 008430 de 1993). It was approved by the Ethics Committee of the Universidad de Antioquia (Act. 16-08-725). After thoroughly explaining the project and clarifying any doubt concerning the research, all subjects signed a written informed consent. The biological material collected was anonymized to ensure the privacy of the individuals.

### The frequency and phenotype of blood T cells

The frequency and phenotype of T cells from blood were determined by flow cytometry. For activation phenotype discrimination, 100 μL of blood was incubated for 30 min at 25°C in the dark with specific antibodies for CD3-PerCp (clone: SK7, Becton–Dickinson BD Biosciences, San Jose, CA), CD4-PE (Clone: SK3, BD), CD8a-Alexa Fluor 700 (clone: OKT8, Thermo Fisher), CD161-APC (clone: DX12, BD), CD38-PE-Cy7 (clone: HIT2, Thermo Fisher) and anti-HLA-DR-FITC (clone: L243, Thermo Fisher). Erythrocytes were lysed with 1× lysing solution (BD, San Jose/CA, USA) incubating for 20 min at 25°C. Cells were washed twice with PBS (Sigma-Aldrich) and then fixed with 1% paraformaldehyde.

For discrimination of regulatory T cells (Treg) and CD161^+^CD4^+^ T cell phenotype (Th17-like [26]), 100 μL of blood was incubated for 30 min at 25°C in the dark with specific antibodies for CD3-PerCp (clone: SK7, BD), CD4-PE (Clone: SK3, BD), CD161-APC (clone: DX12, BD) and CD25-PE-Cy7 (clone: BC96, Thermo Fisher). Erythrocytes were lysed with 1× lysing solution (BD) incubating for 20 min at 25°C. After, the cells were permeabilized and fixed using the Foxp3/Transcription Factor Staining Buffer Set (Thermo Fisher), following the intracellular staining with anti-FOXP3-FITC (clone: PCH101, Thermo Fisher) for 30 min at 4°C. Then, cells were washed twice with PBS (Sigma-Aldrich) and fixed with 1% paraformaldehyde. At last, we estimated the balance in Treg and Th17-like cells using a ratio.

All preparations were stored at 4°C until the acquisition in the LSR Fortessa cytometer (BD). Acquisitions were performed in the BD FACSDiva 6.1.2 version and the data analysis in FlowJo versión 10.4 (Tree Star, Inc, Ashland, OR, USA). Fluorescence minus one control was used to define positive thresholds. The lymphocyte gate was selected by the side (SSC) versus forward (FSC) light scatter, and dead cells were excluded. The CD4^+^ or CD8^+^ T cells were gated within the CD3^+^ region.

The characterization of Treg cells was performed by evaluating the expression of the CD25 and FOXP3 markers in the CD4^+^ LTs. Th17-like cells were determined by the expression of the CD161^+^ in CD4^+^ T cells. Likewise, the activation level was determined by the co-expression of HLA-DR and CD38 in both subsets.

### Microbiota sequencing

DNA was extracted from feces using the stool DNA isolation kit (Norgen Biotek, Thorold, ON, Canada). DNA sequencing and analysis were performed. Briefly, after quality controls assessments, the DNA was sequenced as previously described [27], using the Illumina MiSeq platform. The sequenced data processing was performed following the Standard Operating Procedure (SOP) using MOTHUR software package (v 1.39.5). Quality and specificity reads were assessed, and low quality and nonspecific reads were cleaned with the screen.seqs_command. Operational Taxonomic Units (OTUs) were defined at 97% and were taxonomic assigned using the RDP reference database sequences as a guide [28] (S1 File). Singletons and OTUs, identified as both chloroplasts and mitochondria, were discarded.

### Statistical analysis

The compositional data obtained were normalized using Center Log Ratio (CLR) transformation, and the characterization of microbial community alpha diversity was assessed by the sample’s richness. To visualize differences in microbial community structures based on genera abundances, θ_yc_ dissimilarity matrices were generated from OTU tables, which were obtained with MOTHUR, subsequently observed to Principal Coordinate Analysis (PCoA), and samples were compared through Permutational Multivariate Analysis of Variance (PERMANOVA), performed using Microbiome Analyst [29,30]. In addition, correlations in genera microbial of fecal microbiota samples were calculated using the Spearman test implemented in Microbiome Analyst, following literature recommendations [31]. According to bivariate normality assumption through the Shapiro–Wilk test, parametric unpaired t-tests or non-parametric Mann-Whitney U tests were used to compare the phenotype of blood T cells among the studied groups. A two-tailed p-value <0.05 was considered statistically significant. The statistical tests were performed using the GraphPad Prism V.7. (GraphPad Software, San Diego, CA, USA).

## Results

### The composition of intestinal microbiota in HESN is different from HC but similar to HIV+

We assessed the fecal microbiota of 25 individuals belonging to three groups: HESN (n = 6), HC (n = 9), and HIV+ (n = 10). We asked about the food preference and the intake of different animal protein such as chicken, meat and fish and differences were not observed among the three cohorts of individuals. Likewise, regarding physical activity in all individuals, differences were not highlighted. After bioinformatics analyses, the number of clean sequences of the fecal samples had a median of 29,589 sequences per sample. The sequences obtained were representative of the microbial communities, and sequence numbers were sufficient to characterize the study population, indicated by the rarefaction curves (S1 Fig).

**Fig 1:**
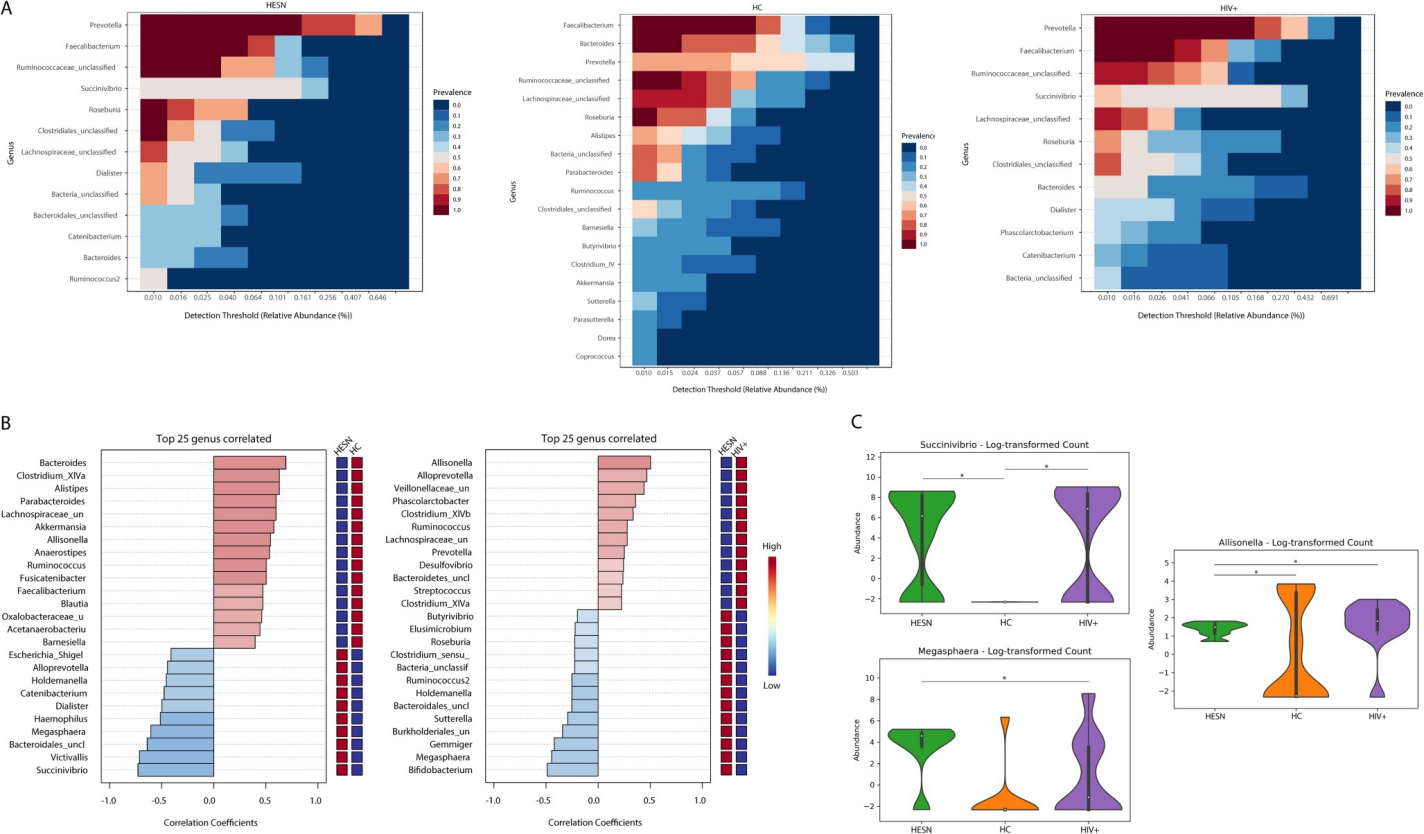
Specific microbial communities in HESN fecal microbiota compared to HC. Genera abundance and prevalence in HESN, HIV+, and HC (**A**) are shown. (**B**) presents the top 25 genera correlated between HESN and HC and HESN and HIV+. Finally, (**C**) shows the main genera correlated in previous analyses performed using Spearman correlation with statistical significance *(r>0.50 and p<0.05).

The composition of microbial communities was assessed in each group. Although we identified common genera of Firmicutes such as *Faecalibacterium* and *Roseburia* genus with similar abundance in the fecal microbiota in all groups analyzed (Fig 1A), microbiota in HESN was different compared to HC. Still, it exhibited a more remarkable similarity with the one observed in HIV+ (Figs 1A–1C and S2), including the family abundance (S3 Fig). Indeed, we observed a similar abundance of genera such as *Prevotella*, *Dialister*, and the Proteobacteria *Succinivibrio* genus shared in HESN and HIV+. In contrast, we observed a lower abundance of *Prevotella* and *Dialister* genera and a higher abundance of Bacteroides in HC compared to HESN and HIV+ (Fig 1A).

Moreover, many genera were not common in HESN and HC; *Succinivibrio* (p = 0.0021), *Victivallis* (p = 0.0029), and *Megasphaera* (p = 0.017) were more significantly enriched in HESN than HC, *Bacteroides* (p = 0.0042), *Clostridium XIVa* (p = (0.011), *Alistipes* (p = 0.011), and other genera from Firmicutes, Bacteroidetes, and some Vellionellales as *Allisonella* (p = 0.036), were preferentially present in HC, but absent in HESN (Fig 1B). However, HESN and HIV+ seem to have a similar microbiota composition since only *Allisonella* (p = 0.047, Fig 1B and 1C) was significantly enriched in HIV+, but not in HESN, suggesting an association of these genera with HIV-1 infection. In this analysis, we observed *Megasphaera* (p = 0.017), and *Allisonella* (p = 0.036) genera being over- and under-represented in HESN when compared to both HC and HIV+, respectively (Fig 1B and 1C).

### Higher richness in the fecal microbiota of HESN

**Fig 2:**
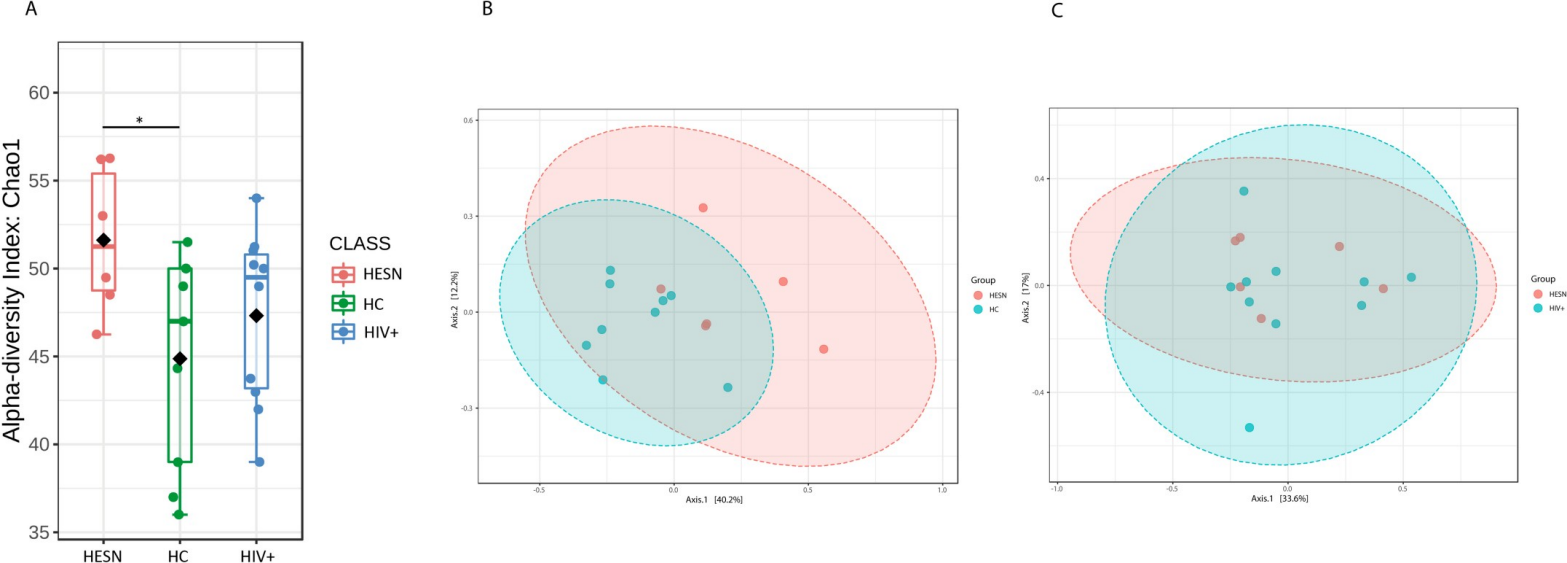
Higher diversity in HESN and similarities with HIV+. (**A**) Microbial community diversity, assessed by the sample’s richness with Chao1 index (alpha diversity) by T-test statistical (p = 0.070). (**B**) Comparison between samples in HESN and HIV+. (**C**) Comparison between samples in HESN and HC, by Principal Coordinates Analysis (PCoA), and using Permutational Multivariate Analysis of Variance (PERMANOVA) in statistical analysis (p = 0.011).

The alpha diversity was assessed using the Chao1 index, a richness indicator. We found a significantly higher richness in HESN than in HC (p = 0.040), whereas there were no differences between HESN and HIV+ (Fig 2A). Likewise, when comparing the microbiota composition among the three groups, through beta diversity analysis, we found that the fecal microbiota of HESN was not clustered with HC, being indeed significantly different between them (p = 0.006, Fig 2B). Additionally, HIV+ and HESN show similar clustering (p = 0.754, Fig 2C).

Altogether these results suggest that fecal microbiota in HESN share features with HIV+. In contrast, fecal microbiota in HESN is substantially different compared to HC, despite none of them harboring the virus. However, an exclusive presentation of some genus in HESN microbial composition was not observed in the analysis.

### Influence of Treg and Th17-like cells in the composition of fecal microbiota

**Fig 3:**
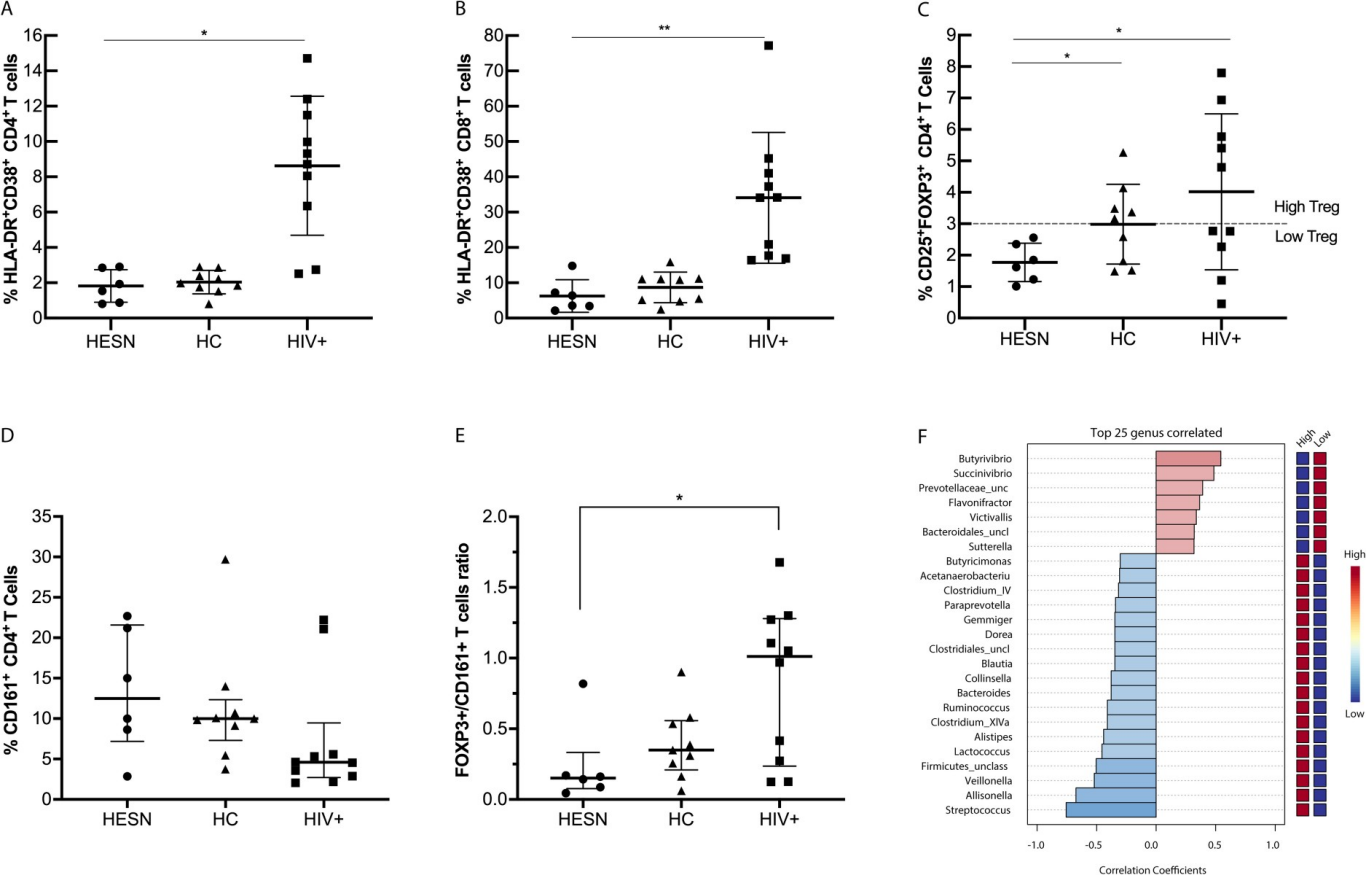
Lower percentage of Treg cells in HESN and influence of immunological characteristics on fecal microbiota composition. The percentage of CD38^-^ and HLA-DR^-^expressing CD4^+^ T cells (**A**) and CD8^+^ T cells (**B**) is shown. The percentage of Treg cells (**C**), Th17-like (CD161^+^CD4^+^) T cells (**D**), and the ratio of Treg/Th17-like cells in each group (**E**) are presented. The statistical analysis among groups was estimated using the Mann-Whitney U test. (**F**) The top 25 genera correlated with a high (>3%) or low (<3%) percentage of Treg cells on fecal microbiota composition is shown. (statistical analysis were performed using Spearman test; p-value <0.05). *p-value <0.05. **p-value <0.01.

Subsequently, we wonder if fecal microbiota varied depending on some immunological characteristics. Accordingly, we compared the percentage of hyperactivated T cells among the groups, as previously reported [32]. As expected, HIV+ had a significantly higher percentage of activation of T cells (8.63% CD4^+^, and 34.1% CD8^+^) than HESN (1.82% CD4^+^, and 6.22% CD8^+^), and HC (2.03% CD4^+^, and 8.69 CD8^+^). Moreover, no differences were observed between HESN and HC in any of the cell subsets evaluated, and both groups maintained low levels of T cell activation (Fig 3A and 3B).

Furthermore, we assessed the percentage of CD4^+^CD25^+^FOXP3^+^ regulatory T cells (Treg), and we found that HESN had a lower percentage of Treg cells (1.77%) compared to the HC group (2.98%) and the HIV+ group (4.02%) (p = 0.12 and p = 0.0065 respectively, Fig 3C). Additionally, we assessed the percentage of Th17-like cells by CD4^+^CD161^+^ phenotype, and although HESN seems to have higher Th17-like percentages than HIV+ and HC, there were no significant differences among the groups (Fig 3D). Finally, we explored potential differences among the groups in the balance of regulatory and inflammatory T cells using the ratio Treg/Th17-like cells. We found that HESN had a significantly lower Treg/Th17-like ratio than HIV+, whereas no statistically significant differences were observed between HESN and HC (Fig 3E).

Since we observed significant differences between HESN and other groups in Treg percentage, being a relevant immunological characteristic decreased in HESN, we classified the percentages of Treg cells according to the median percentage of Treg cells of HC as previously assessed [33]. Accordingly, we defined Treg percentage as high (>3%) or low (<3%), and then, we compared fecal microbiota composition in clustered samples by a high and low Treg cells percentage. We found that the genus *Butyviribrio* was enriched in subjects with low Treg (r = 0.56 p = 0.029, Fig 3F), a cluster represented by all HESN and half of HC (Fig 3C). Besides, we could highlight the positive correlation of the *Succinivibrio* genus with low Treg (r = 0.48, p = 0.066, Fig 3F), a genus that we have found enriched in HESN (Fig 2). Subsequently, we observed negative correlations between low Treg and *Streptococcus* (r = -0.75, p = 0.001), *Allisonella* (r = -0.67, p = 0.006), and *Veillonella* (r = -0.52, p = 0.046) (Fig 3F). We also explored the correlations between the higher and lower ratio of Treg/Th17-like cells and fecal microbiota composition among groups, but no differences were observed (S4 Fig).

Although these findings suggest that Treg might influence microbiota composition, its impact seems not to be sufficient to affect microbial diversity and abundance, at least in HC, since no differences were observed in alpha and beta diversity when we compared microbiota between HCs with low and high Treg percentages (S5 Fig).

### The abundance of some genera is shared between HESN and HIV+

**Fig 4:**
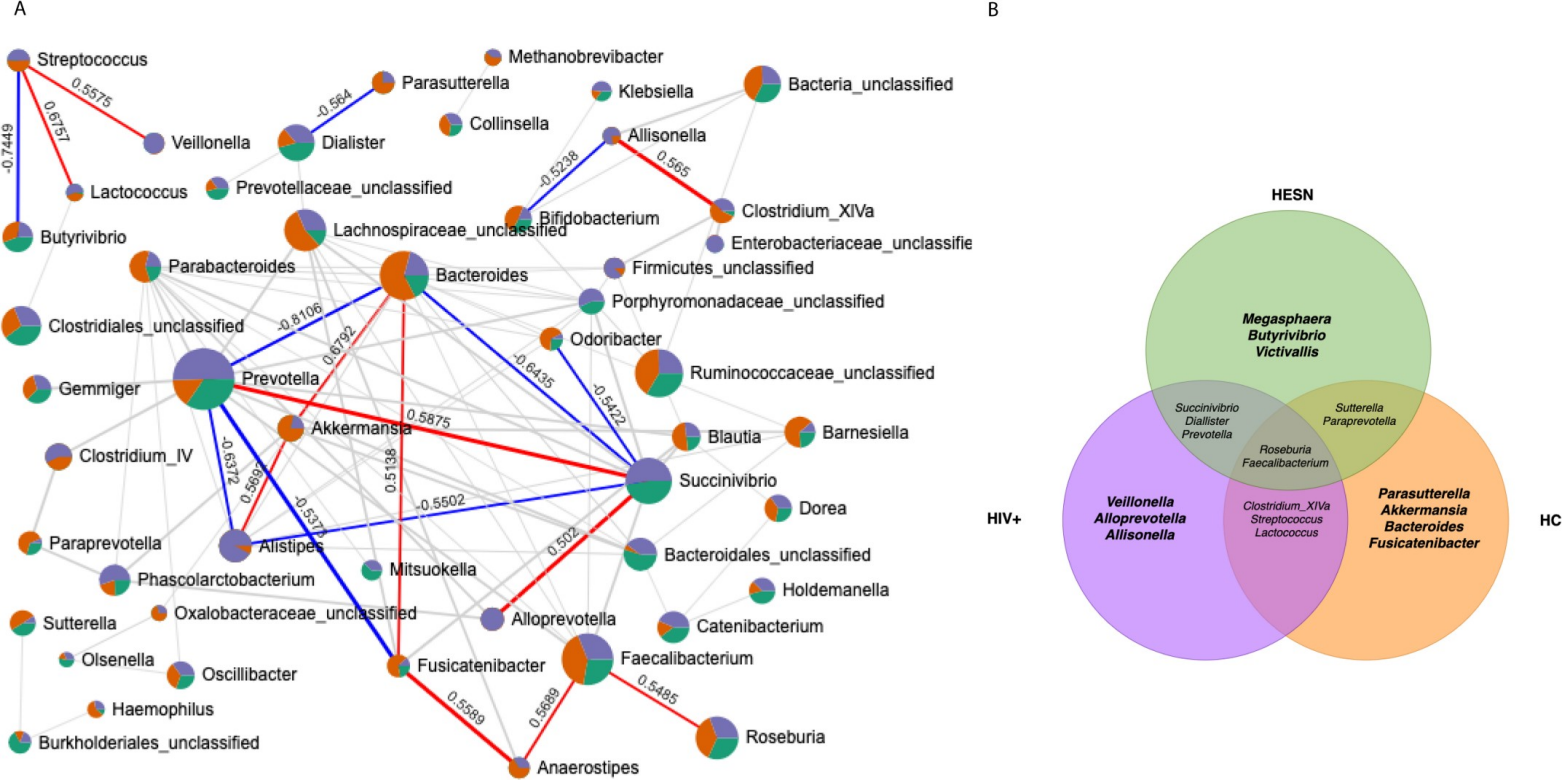
The Abundance of *Prevotella* and *Succinivibrio* in fecal microbiota composition of HESN. (**A**) shows the correlation network of the most abundant genera in HESN (green), HIV+ (purple), and HC (orange), and interaction between microbial community’s genera; the circle’s size represents the abundance of genera in microbial composition. The correlations were estimated using Spearman correlations (p<0.05, r>±0,5), the most significant bacteria are highlighted as positive (red) or negative (blue) correlations. Venn diagram (**B**) shows the main genera of each group studied as well as those shared between them.

The fecal microbiota in HESN subjects is particular in its composition. However, the enrichment of specific genera such as *Prevotella*, *Succinivibrio*, and *Dialister* in both HESN and HIV+ is noteworthy. We evaluated the abundance and influence of some genera in the microbial communities through a network of positive or negative correlations among them in all groups. We found genera of great interest in the context of HIV exposure, especially the similar abundance from *Prevotella* and *Succinivibrio* both in HESN and HIV+, and the positive correlation observed between these genera (Fig 4A). These genera interact, allowing the consolidation of a particular profile in HESN individuals, with characteristics shared with HIV+. We highlighted the positive correlations between *Streptococcus* and *Veillonella* (p = 0.0038), and the negative ones with *Butyrivibrio* (p≤0.0001), genera associated with variations in Treg percentage (Figs 3F and 4A). Furthermore, the network analysis also showed a negative correlation between *Bifidobacterium* and *Allisonella* (p = 0.0093), genera related to HESN and HIV+, respectively (Fig 4A).

In summary, we identified some genera that, although not exclusive, are enriched in the intestinal microbiota of a specific group of subjects and are part of the most outstanding characteristics in the microbiota of each one. We outlined in a Venn diagram the genera characterizing the microbiota profile in the three studied groups (Fig 4B). Specifically, we found that *Megasphaera*, *Victivallis* and *Butyrivibrio* are enriched in the microbiota composition in HESN. On the other hand, *Bacteroides*, *Akkermansia*, and *Parasutterella* were clustered as healthy microbiota composition, and *Veillonella*, *Allisonella*, and *Alloprevotella* were clustered as genera highlighted in HIV+ subjects. Other genera were highlighted to be shared in some groups but absent in the other, for instance, the case of *Prevotella*, *Succinivibrio*, and *Dialister* shared in HESN and HIV+, but absent in HC. Finally, other genera such as *Faecalibacterium* and *Roseburia* were identified as very common microbiota in the three studied groups (Fig 4B).

## Discussion

The mechanisms of natural resistance to HIV have been a subject of interest. We aimed to characterize the fecal microbiota profile of a small group of HESN from a Colombian cohort compared to the microbiota of HC and HIV+ progressors. Previous studies have previously studied the microbiota in cervicovaginal mucosa in heterosexual women as a correlate of protection in HIV-exposed sex workers from Africa [21]. However, to the best of our knowledge, this is the first study describing the characteristics of the fecal microbiota in a group of HESN in Latin American individuals. These preliminary results suggest that exposure to HIV in seronegative individuals seems to confer specific composition to their fecal microbiota. Indeed, HESN individuals have a particular profile characterized by bacterial genera such as *Megasphaera*, *Succinivibrio*, and *Victivallis*, as well as by a high richness composition.

Interestingly, many of the bacteria found in HESN were common with HIV+ subjects. In fact, there were no significant differences between both groups in alpha and beta diversity analyses of fecal microbiota. Remarkably, microbiota composition in HIV-infected individuals stands out by increased numbers of Proteobacteria *Succinivibrio* and *Klebsiella; Veillonellaceae* pathobionts such as *Dialister*, *Allisonella*, and *Veillonella*, as well as by enrichment of *Prevotella* and *Alloprevotella*, as previously described worldwide [10–12].

Most of these genera (except for *Allisonella*, *Alloprevotella*, and *Veillonella*) were shared with HESN. Thus, we hypothesized that the microbiota in HESN could be acquired due to cohabiting and experiencing unprotected sexual intercourse with HIV seropositive partners [19,34,35]. It has been shown that cohabiting with a person [36], experiencing certain types of sexual relations [37], and sharing with pets [38] causes similar composition of microbial communities.

In this study, the genera *Allisonella*, *Alloprevotella*, and *Veillonella* were almost absent in HC and HESN but predominant in HIV+, and thus related with infection. Why these bacteria are not transmitted from HIV+ to HESN remains to be elucidated. *Alloprevotella* was reported in men who have sex with men and was even related to an early diagnosis of anal precancerous lesions [37]. *Allisonella* has also been reported in lower genital tract microbiota from HIV-infected women [39].

Here, *Succinivibrio* was shared in fecal microbiota between HESN and HIV+, which is striking because this genus has been associated with infection control in HIV-1 elite controllers, possibly because it could influence a metabolic profile (fatty acid metabolism, lipid biosynthesis, and tryptophan pathways) that may contribute to HIV-1 control [35]. Recently, an increase in *Succinivibrio* and *Megasphaera* have been described in infected patients under antiretroviral therapy [40]. In addition, the *Megasphaera* genus has been found in high proportion in healthy vaginal microbiota, especially in the genital tract [41]. It is important to note that most HESN in this study were female, and their HIV-infected partners were on ART at sampling, which could explain the enrichment of these genera in HESN.

It is known that the production of favorable short-chain fatty acids (SCFA) by some bacteria has anti-inflammatory, antitumorigenic, and antimicrobial effects, maintaining gut integrity and immune homeostasis [42]. Interestingly, genera enriched in HESN such as *Succinivibrio*, *Megasphaera*, *Butyrivibrio*, and *Victivallis* are known SCFA-producers [41–44], suggesting a larger SFCA pool in HESN than HIV+ subjects. However, the relevance of these genera in HESNs remains to be fully described.

HESNs showed higher species richness than HCs when evaluating the Chao-1 index, being enriched in microbial genera that were not very abundant but particular of these individuals. Changes in microbial diversity or species richness are characteristics that other authors have reported in HIV-infected individuals [7], in whom bacterial alpha diversity was associated with lower expression of different biomarkers (soluble or cellular) of immune stimulation or T cell dysfunction [12,45].

HIV-infected patients, particularly during disease progression, show gut microbiota alteration and a massive destruction of Th17 cells and expansion of dysfunctional Treg cells, which induces an imbalance in the Treg/Th17 ratio, allowing microbial proliferation, dysregulation, and dysbiosis in the gut [46,47]. Althoug some authors have found an increase in Treg percentage associated with natural resistance to HIV-1 infection in commercial sex workers [48], we observed that low Treg percentage is a common characteristic in our HESNs coming from serodiscordant couples, allowing us to especulate that type of exposure could diferentaially modulate Treg subset. Interestingly, the subjects with low Treg percentage had an enrichment of the genus *Butyrivibrio*. However, when Th17 cells were analyzed alone, they were not significantly relevant in our study. A decrease of relative abundance of Clostridia, including *Butyrivibrio*, has been found in HIV-related microbiota signatures consisting of lower diversity, especially in those patients with severe immunodeficiency [49]. We found *Butyrivibrio* is enriched in subjects with low Treg percentage, while *Streptococcus* and *Allisonella* are associated with higher Treg percentage. However, Treg percentage alone was insufficient to explain the microbiota profile in this group since no differences in alpha and beta diversity analyses between subjects with high and low Treg percentage were found. Nevertheless, additional studies are needed to define Treg cells and Treg/Th17 ratio role in modulating microbiota and HIV resistance.

Although these results are interesting, they need to be interpreted with caution since this study is limited by the small sample size and the fact that HIV+ partners of HESN individuals were not studied. However, the expansion of antiretroviral therapy coverage worldwide has reduced the viral load and thus the viral exposure in serodiscordant couples, making it challenging to identify a larger number of HESNs.

Also, the microbiota variability could be influenced because the individuals included in the study came from two different cities and because food intake was not controlled. Nonetheless, we applied surveys regarding diet, medication consumption, and recent diseases, but we did not see any notable differences among the sampled subjects. Being aware of these limitations and thus requiring confirmation in bigger cohorts, the evidence described in this study suggests that the microbiota in HESN has a characteristic profile, similar to that of HIV+, most likely because HESNs cohabit with their HIV+ partners.

## Supporting information

S1 Fig Rarefaction curves of the sequences obtained. (TIF)

S2 Fig Heatmap of particular profiles in the HESN, HC, and HIV+ in microbial genera. (TIF)

S3 Fig Characterization of the abundance of microbial communities according to genera and families in the fecal microbiota of HESN and HC. (TIF)

S4 Fig Influence of Treg/Th17-like cells ratio in top genera correlated in fecal microbiota. (TIF)

S5 Fig Characterization of microbial composition in the fecal microbiota of HC, with low and high Treg cells percentages. (TIF)

S1 File Sequences database and metadata used for the analysis. (XLSX)
