# Supplementary figures and images for: A specific structure and high richness characterize intestinal microbiota of HIV-exposed seronegative individuals
Source: PLoS One. 2021 Dec 2;16(12):e0260729. doi: 10.1371/journal.pone.0260729 (PMC8638974; doi:10.1371/journal.pone.0260729)

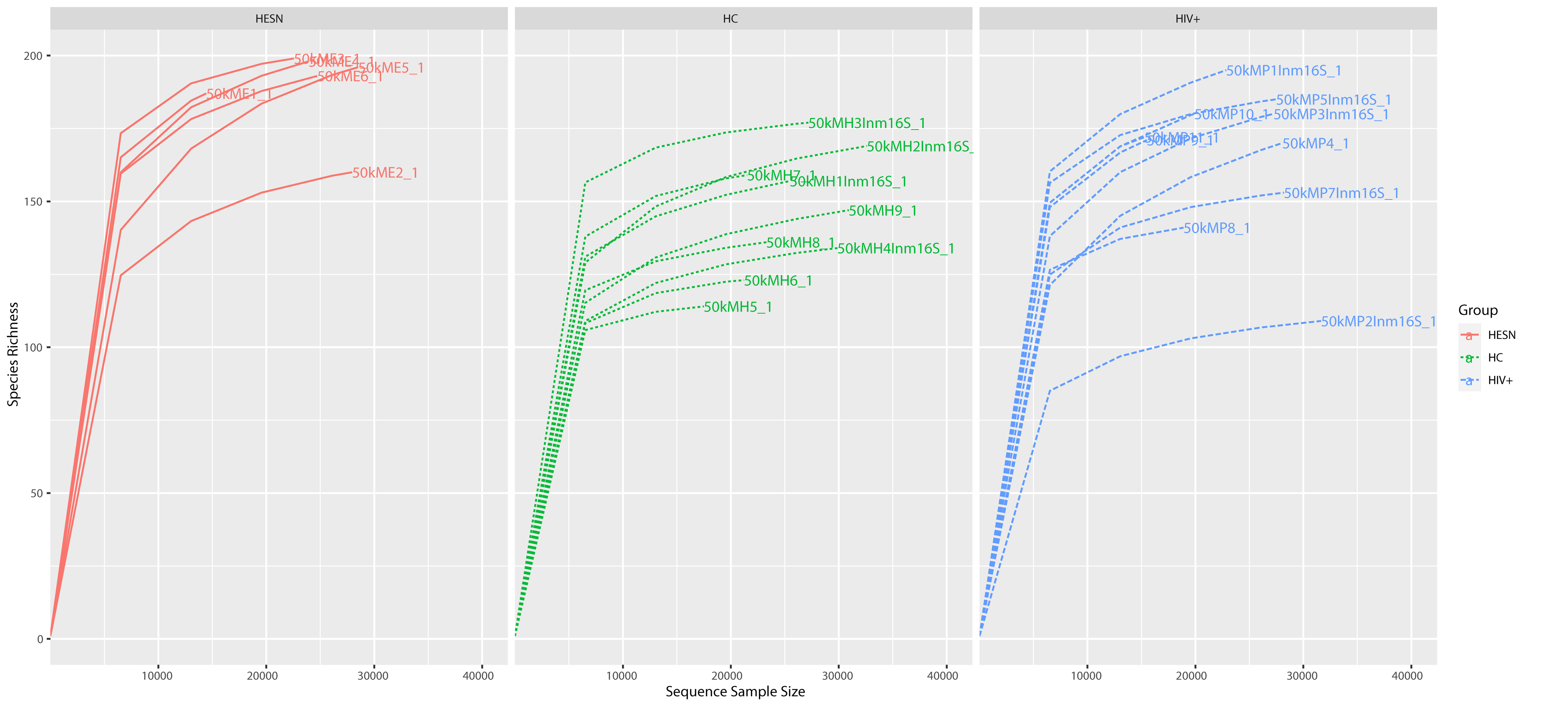

Supplement: S1 Fig — (TIF) [file pone.0260729.s001.tif]

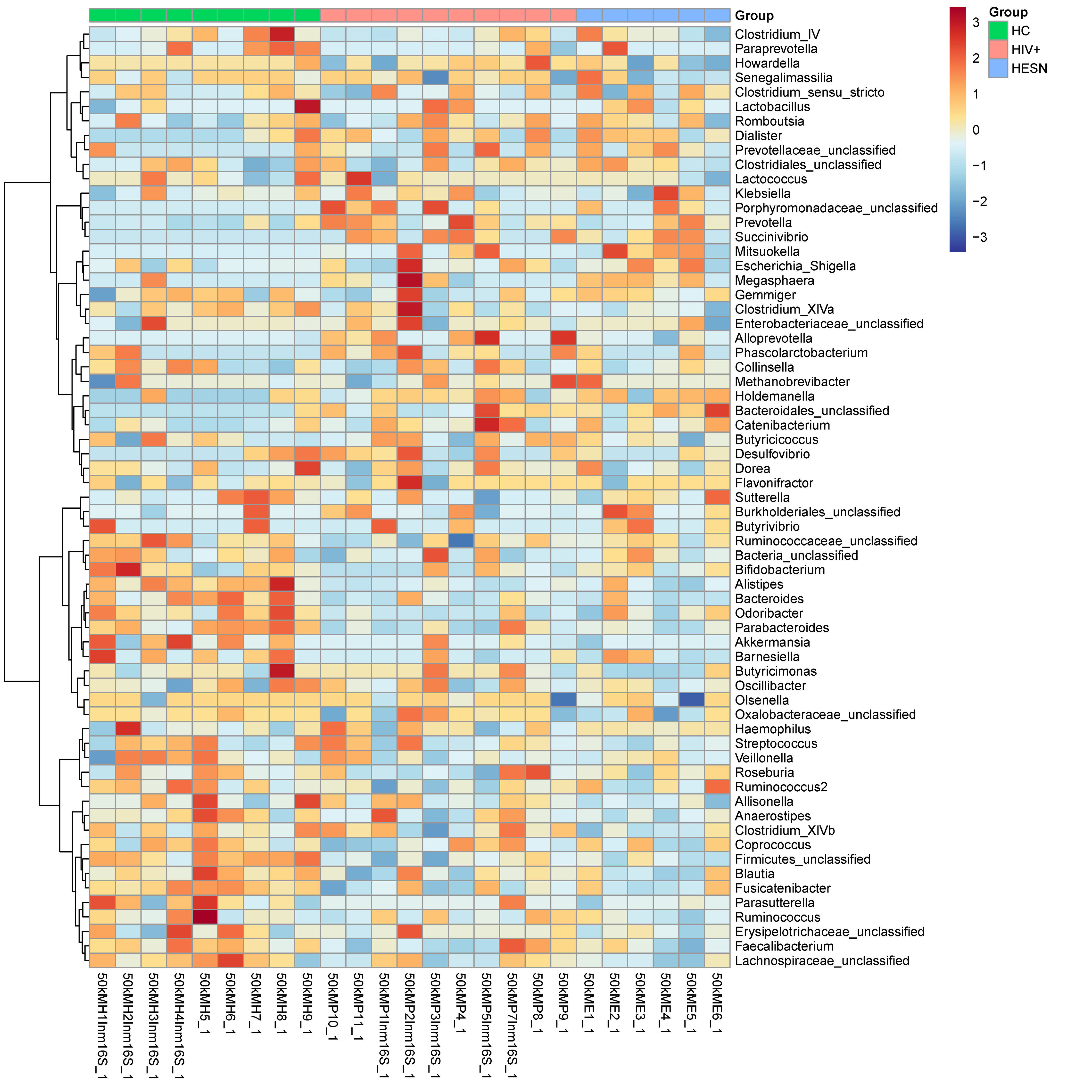

Supplement: S2 Fig — (TIF) [file pone.0260729.s002.tif]

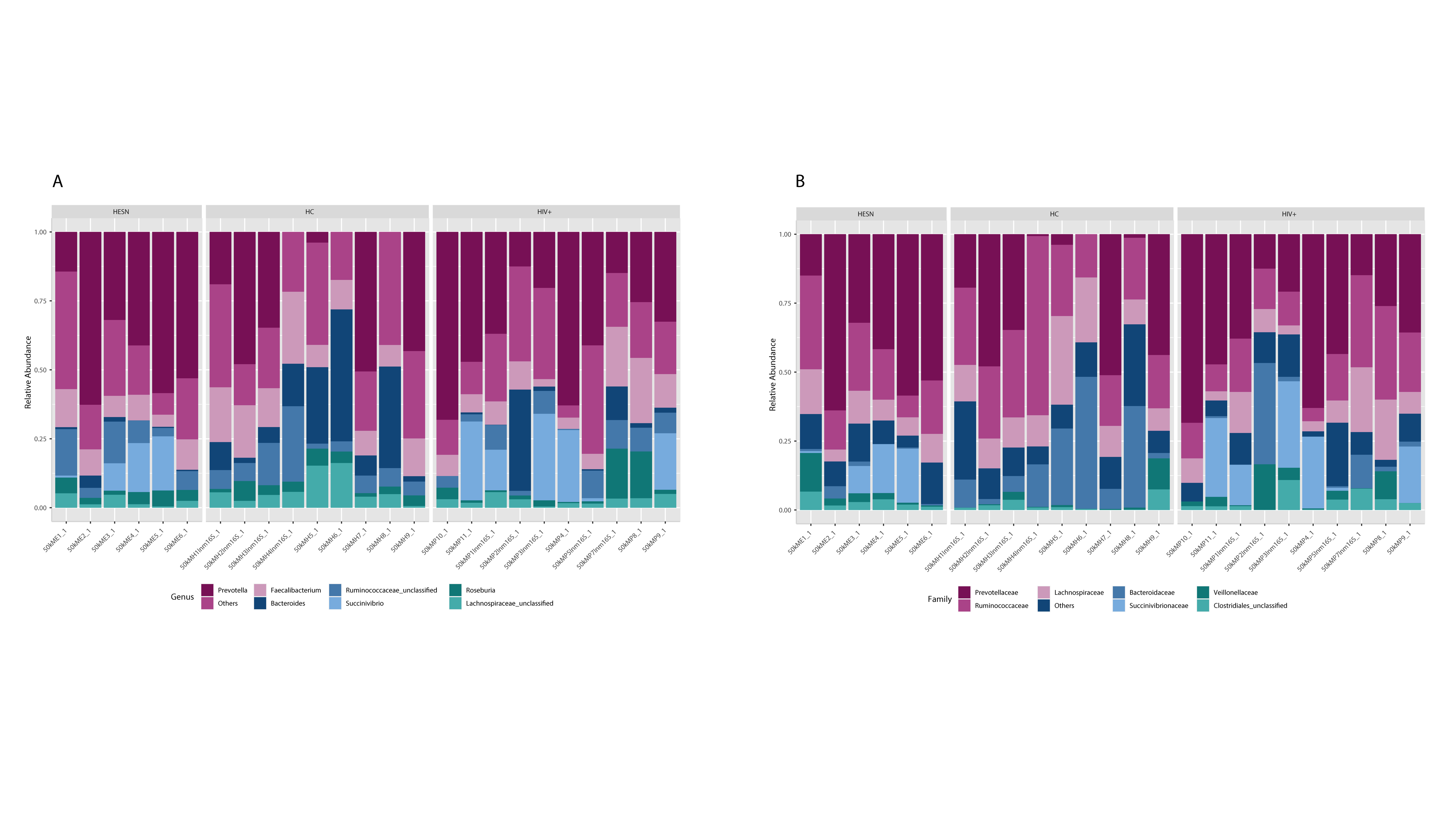

Supplement: S3 Fig — (TIF) [file pone.0260729.s003.tif]

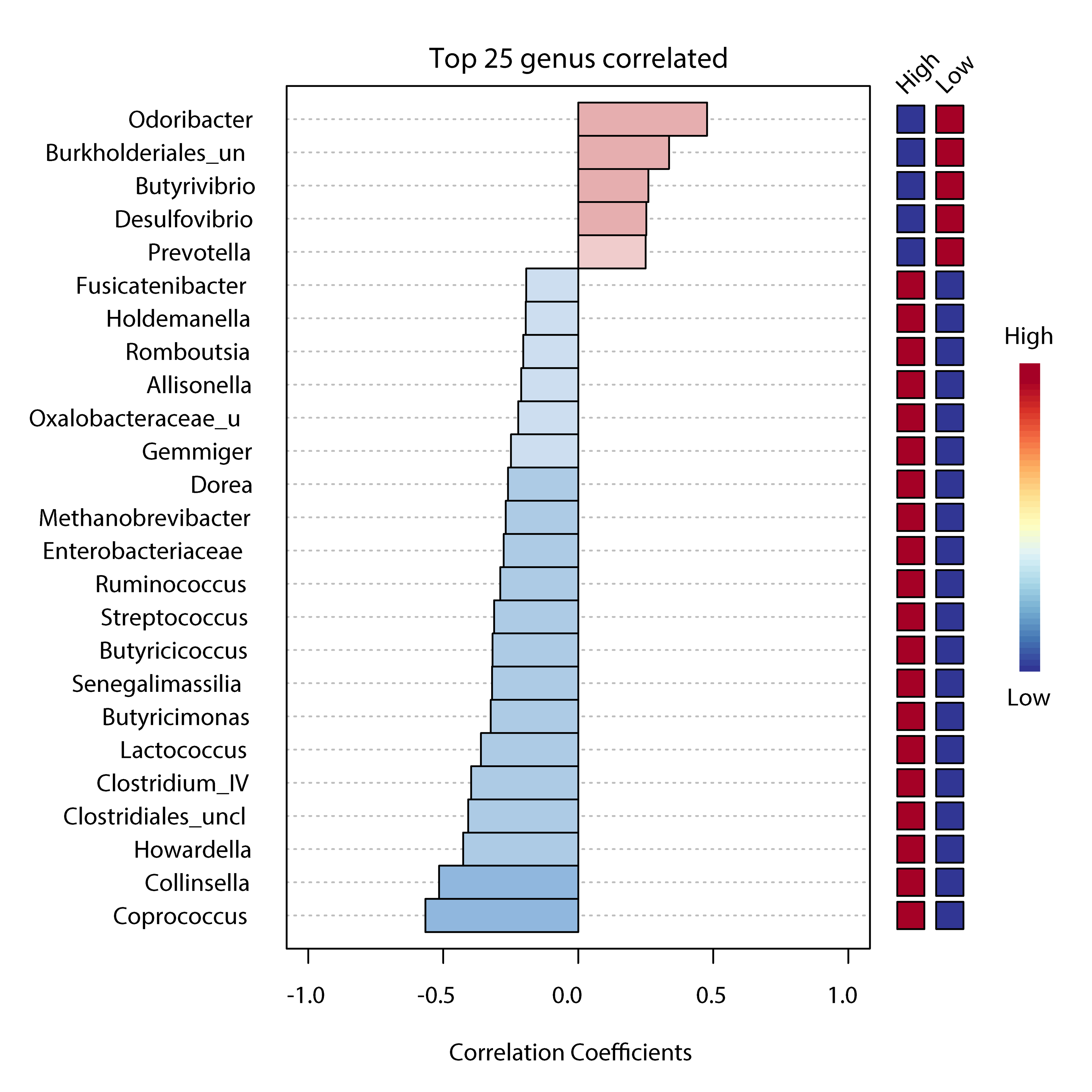

Supplement: S4 Fig — (TIF) [file pone.0260729.s004.tif]

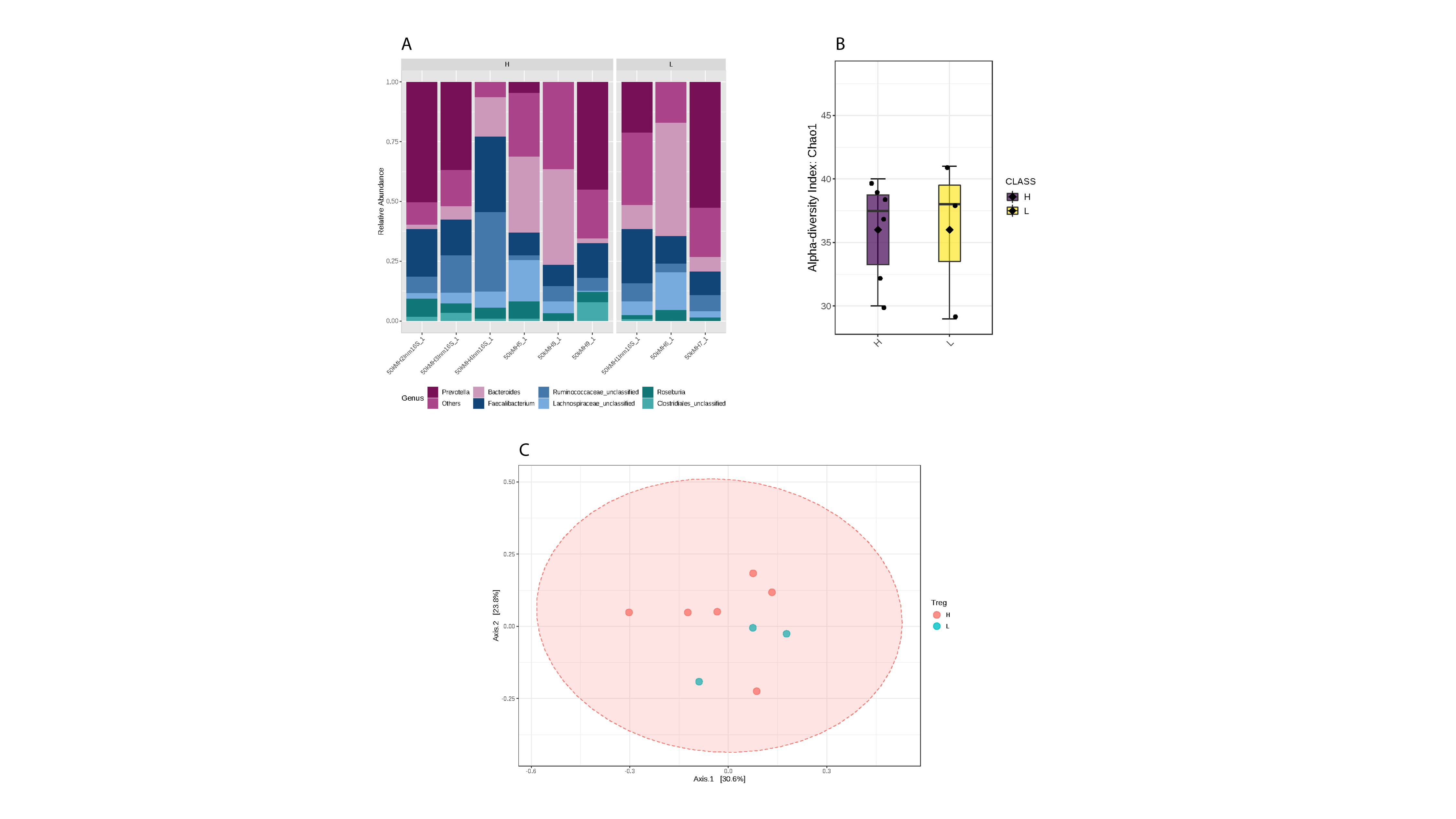

Supplement: S5 Fig — (TIF) [file pone.0260729.s005.tif]
